# Effectiveness and tolerance of enteral nutrition in critically ill patients with COVID-19

**DOI:** 10.1017/S0007114524002666

**Published:** 2024-12-14

**Authors:** Elizabeth Pérez-Cruz, Salvador Ortiz-Gutiérrez, Jorge Alberto Castañón-González, Yuritzy Luna-Camacho, Jessica Garduño-López

**Affiliations:** 1 Department Metabolic Unit and Nutritional Support, Hospital Juárez de México, México City, Mexico; 2 Obesity Clinic, Hospital Juárez de México, México City, Mexico; 3 National Autonomous University of Mexico, México City, Mexico; 4 Department Adult Intensive Care Unit, Hospital Juárez de México, México City, Mexico

**Keywords:** SARS-CoV-2, COVID-19, Enteral nutrition, Nutrients, Prone ventilation, Diarrhoea

## Abstract

This study compared the efficacy and tolerability of three enteral formulas in critically ill patients with COVID-19 who were ventilated and in the prone position: (a) immunomodulatory (IMM), (b) ω3 and (c) maltodextrins (MD). Primary outcome was the percentage of patients who received both 80 % of their protein and calorie targets at 3 d after enrolment. Secondary, mechanical ventilation-free time, ICU mortality and markers of nutritional status. Tolerance of enteral nutrition was evaluated by diarrhoea and gastroparesis rate. A total of 231 patients were included, primary outcome achieved was in ω3 group (76·5 % *v*. 59·7 and 35·2 %, *P* < 0·001) *v*. IMM and MD groups. Mechanical ventilation-free time was longer in ω3 and MD groups: 23·11 (sd 34·2) h and 22·59 (sd 42·2) h *v*. 7·9 (sd 22·6) h (*P* < 0·01) in IMM group. Prealbumin final was 0·203 ± 0·108 g/L and 0·203 ± 0·095 g/L in IMM and ω3 groups *v* 0·164 ± 0·070 g/L (*p* < 0·01) MD group. Transferrin were 1·515 ± 0·536 g/L and 1·521 ± 0·500 g/L in IMM and ω3 groups *v* 1·337 ± 0·483 g/L (p < 0·05) MD group. Increase of lymphocytes was greater in ω3 group: 1056·7 (sd 660·8) cells/mm^3^ *v*. 853·3 (sd 435·9) cells/mm^3^ and 942·7 (sd 675·4) cells/mm^3^ (*P* < 0·001) in IMM and MD groups. Diarrhoea and gastroparesis occurred in 5·1 and 3·4 %, respectively. The findings of this study indicate that enteral nutrition is a safe and well-tolerated intervention. The ω3 formula compared with IMM and MD did improve protein and calorie targets.

The global pandemic caused by the SARS-CoV-2 virus first emerged in Wuhan, China, in late 2019. Subsequently, the WHO has declared the end of a global health emergency, noting that the disease will continue to affect the global population. In recent weeks, there has been a notable increase in the prevalence of the JN.1 variant globally.^(1)^ It has been observed that older adults and patients with co-morbidities such as diabetes mellitus, hypertension and obesity have been the most vulnerable to severe SARS-CoV-2 infection.^(1,2)^ Patients with severe COVID-19 exhibit a prominent systemic inflammatory response, characterised by the release of pro-inflammatory cytokines, including IL-1*β*, IL-6 and TNF-*α*. This cytokine storm induces a severe metabolic alteration, increasing both resting energy expenditure and protein catabolism.^(3)^ Acute respiratory distress syndrome (ARDS) following SARS-CoV-2 pneumonia represents the most severe form of pulmonary compromise and, like that produced by other causes, also leads to an increase in energy expenditure and protein catabolism.^(4)^

The initiation of early enteral nutrition (EN) within 48 h of admission to the intensive care unit (ICU) in haemodynamically stable patients is considered the best practice for the prevention of nutritional and metabolic deterioration.^(5,6)^ Adequate calorie and protein intake have been linked with enhanced outcomes and a reduction in the number of days spent on mechanical ventilation among critically ill patients. On the other hand, the accumulation of caloric deficits and negative protein balances have been linked with an increased incidence of complications, particularly healthcare-associated infections, a longer hospital stay and a higher in-hospital mortality rate.^(7–9)^ Even though indirect calorimetry remains the gold standard for assessing energy expenditure, the European Society for Clinical Nutrition and Metabolism recommends its use, provided the sterility of the measurement system is ensured.^(10)^ Other societies, such as the American Society for Enteral and Parenteral Nutrition and the Australian Society for Enteral and Parenteral Nutrition, report that indirect calorimetry could increase the risk of infection in healthcare workers.^(11,12)^ In addition to a reduction in the number of medical staff and a high level of patient demand, the use of this method was also limited. The European Society for Clinical Nutrition and Metabolism recommends a contribution of 20 kcal/kg per d during the acute phase and a progressive increase to 80–100 % of energy requirements and a protein goal of 1·3 g/kg per d.^(10)^ In contrast, the American Society for Enteral and Parenteral Nutrition recommends a protein intake of 1·2–2·0 g/kg per d and 15–20 kcal/kg per d in critically ill patients with COVID-19.^(11)^

The type of enteral formula and the optimal amount of nutrition administered are controversial in patients with ARDS, and significant differences in nutritional treatment have been noted.^(5,6,13)^ The recommendation based on expert consensus is to use a standard polymeric formula when starting EN. However, immunomodulatory (IMM) enteral formulations in some meta-analyses have suggested beneficial effects in reducing infection, hospital stay and duration of mechanical ventilation.^(10)^ In the case of ARDS caused by SARS-CoV-2, being a new viral disease, there is a lack of information on nutritional support practices and on the characteristics and benefits of different feeding formulas, a situation that needs to be improved, as SARS-CoV-2 infection continues to impact healthcare resources worldwide. Furthermore, the prone position has been widely utilised in patients with severe hypoxaemia, including those with ARDS caused by SARS-CoV-2. However, the evidence regarding the safety of this approach and the potential increased risk of feeding intolerance remains inconclusive.^(14)^ We hypothesised that an enteral feeding formula IMM in COVID-19 patients ventilated in ICU and prone position would be associated with achieving an optimal amount defined as 80 % of the 24-h protein and calorie target. Therefore, this study aimed to provide an overview of the mode of nutrition therapy by comparing the fulfilment of caloric and protein targets; additionally, a secondary aim was to determine if any formula enteral may impact mechanical ventilation-free time, mortality and markers of nutritional status.

## Methods

### Study design and population

**Figure 1.**
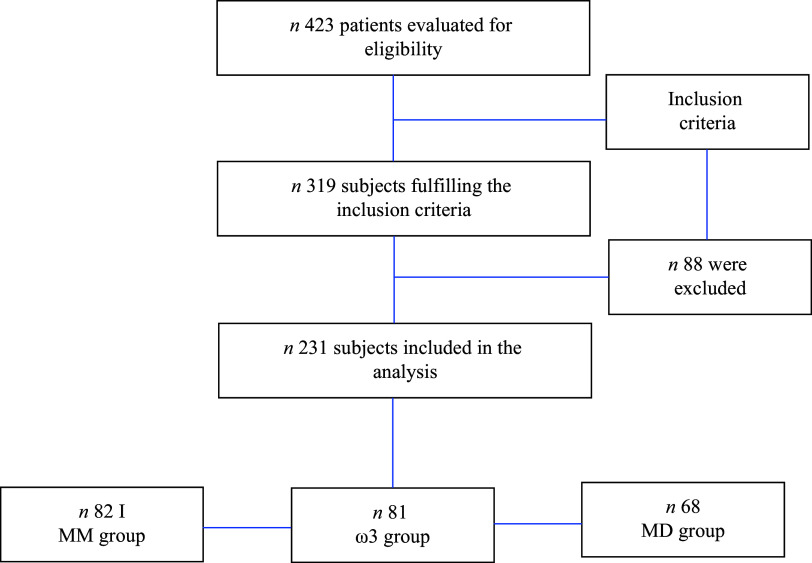
The flow chart of recruitment.

A prospective cohort study was conducted on consecutive adult patients with severe SARS-CoV-2 infection admitted to the multidisciplinary ICU of a third referral hospital in Mexico City between March 2020 and March 2022. All patients with SARS-CoV-2 requiring invasive mechanical ventilation with an expected duration of > 72 h in the prone position and receiving EN within the first 48 h after admission were included. The patients were diagnosed with SARS-CoV-2 pneumonia according to the WHO criteria, including characteristic symptoms of ARDS: dyspnoea, tachypnoea, decreased oxygen saturation, with oxygen requirement of 6 l/min and detection of SARS-CoV-2 virus by real-time RT-PCR assays. Patients requiring exclusive parenteral nutrition (PN), those receiving PN for less than 72 h, those who died within 24 h of ICU admission and those diagnosed with chronic kidney disease KDIGO IV–V were excluded (Fig. 1). The required sample size was calculated using a 95 % confidence level and a margin of error of 4 %, based on the annual admissions to the ICU in the previous year. The resulting sample size was 231 total subjects, who were consecutively enrolled in the study. The institutional ethics committee at a participating centre approved the study protocol (protocol no. 0767/20–1), and the study was conducted in accordance with the Declaration of Helsinki as revised in 2013. Informed consent was waived because we used anonymised retrospective data.

### Data collection

Data were obtained from ICU admission and daily follow-up sheets from the nutritional support unit. Data collected included patient demographics and disease severity, anthropometric measurements, laboratory data (markers of nutritional status), mechanical ventilation-free time and ICU mortality.

#### Demographic variables

The demographic data recorded were sex, age, presence of diabetes mellitus, arterial hypertension, dyslipidaemia, hypothyroidism and chronic lung disease. The Sequential Organ Failure Assessment score and the Acute Physiology and Chronic Health Evaluation II prognostic classification were used to assess the severity of each patient’s illness. The PaO2/FiO2 index was calculated and classified according to the Berlin criteria.^(15)^

#### Anthropometric measurements

The measurements included weight (kg), registered by the Stryker InTouch metabolic beds, and height (m). BMI was calculated, and patients were classified according to the WHO reference ranges.^(16)^ Nutritional risk was assessed using the modified nutrition risk in the critically ill (mNUTRIC) score.^(17)^

#### Laboratory tests

The laboratory tests registered were performed on venous blood according to the standardised methods of the hospital’s central laboratory and included total leucocyte count (cells/mm^3^), neutrophils (cells/mm^3^), prealbumin (mmol/l), transferrin (mmol/l) and albumin (g/dl).

### Nutrition prescription

The use of indirect calorimetry was not feasible due to the high risk of contamination by the dispersion of the SARS-CoV-2 virus through aerosols. Therefore, caloric requirements were determined according to standard protocols, which consist of using the Harris–Benedict equation and adjusting the weight as follows: using current weight for patients with normal BMI (18·5–24·9 kg/m^2^), ideal weight for those with low BMI (< 18·5 kg/m^2^) and adjusted weight for cases with obesity and overweight (BMI > 25 kg/m^2^). An initial stress factor of 1·3 was added to the resulting value in all cases. The protein requirement was initially established at 1·7 g/kg per d; thereafter, both requirements were subsequently adjusted based on clinical evolution, laboratory test results and nitrogen balance. The decision to prone position was made by the intensivist physician based on the severity of ARDS and the patient’s response to initial treatment, with the procedure performed by trained staff following standard unit protocols. Nutrition prescription was initiated by the clinical nutrition specialist physicians as early as possible (within 24–48 h) in the absence of contraindications based on the European Society for Clinical Nutrition and Metabolism and American Society for Enteral and Parenteral Nutrition guidelines for nutritional support in critically ill patients.

### Nutrition therapy

The feeding at admission, prescription, volume prescribed, volume administered and type of enteral formulae were recorded. The three types of enteral formulae that were used were as follows: (a) IMM: isoenergetic, high protein, IMM with arginine, glutamine, branched-chain amino acids, ω3 fatty acids, medium-chain TAG and antioxidants. It provides 1 kcal/1 ml, 32·7 % protein, 47·6 % carbohydrates and 19·7 % lipids (Enterex IMX®, Victus Inc.); (b) ω3: high calorie, high protein, supplemented with ω3 fatty acids, 0·50 g of EPA and 0·21 g of DHA. It provides 1·5 kcal/1 ml, 27 % protein, 33 % carbohydrates and 40 % lipids (Supportan DKN®, Fresenius KABI); and (c) MD: maltodextrins and ω3 and ω6 fatty acids. It provides 0·91 kcal/ml, 20 % protein, 46 % carbohydrates and 34 % lipids (Glucerna®, Abbott) plus a glutamine module with *Lactobacillus reuteri*, which provides 10 g of l-glutamine and 10^8^ CFU of *Lactobacillus reuteri* (Glutapak®, R Pisa).

All patients had a nasogastric/orogastric tube inserted and position was always confirmed with a chest X-ray prior to prone positioning. Enteral nutrition was administered by continuous infusion at an initial rate of 20 ml/h, increased then to 40 ml/h and gradually according to the gastric residual volume until the 80 % caloric target was reached within the first 72 h. If the gastric residual volume was > 500 ml, the infusion rate was reduced, and the EN stopped if necessary. The EN was resumed at an infusion rate of 20 ml/h when the gastric residual volume was ≤ 500 ml. Patients were followed until discharge from the ICU or death. If the patient presented diarrhoea, the infusion rate was decreased to 20–40 ml/h for the next 24 h, and if the symptoms persisted, the EN was stopped. In the case of gastroparesis, intravenous prokinetics (metoclopramide 10 mg three times a day) were prescribed.

### Outcomes

The primary outcome was the percentage of patients receiving both 80 % of the 24-h caloric and protein targets at 3 d after enrolment. Secondary outcomes were mechanical ventilation-free time, ICU mortality and markers of nutritional status (albumin, prealbumin, transferrin and lymphocytes). Tolerance of EN was assessed by the rate of diarrhoea and gastroparesis. Gastroparesis was defined as a gastric residual volume > 500 ml and diarrhoea as more than three watery stools per d for two consecutive days. Nutritional impact was evaluated by the variation of albumin, prealbumin and transferrin measured at baseline and after enrolment.

### Statistical analysis

The baseline characteristics of the groups were analysed using descriptive statistics. Continuous variables are expressed as mean and standard deviation or median and interquartile range (IQR, 25th and 75th percentiles). Categorical data are expressed as frequencies and percentages. Comparisons between groups were made using the *χ* ^2^ or Kruskal–Wallis test for qualitative variables, as appropriate, and the Student’s *t* test for quantitative variables. Differences between repeated measurements within each group were analysed using repeated-measures ANOVA. A significance level of 5 % was used for all statistical tests. Data were analysed using SPSS statistical software for Windows (version 21.00, SPSS Inc).

## Results

A total of 231 patients were included in the study. The demographic characteristics, anthropometric measurements and assessment scales performed on admission to the ICU are shown in Table 1.

**Table 1.** Clinical characteristics according to different enteral formulae (Numbers and percentages; mean values and standard deviations)

Characteristics	Total		IMM group		ω3 group		MD group	
	*n* 231		*n* 82		*n* 81		*n* 68	
	Mean	sd	Mean	sd	Mean	sd	Mean	sd
Age (years)	54·5	12·6	53·0	12·8	52·9	13·6	58·3	11·1^*^
	*n*	%	*n*	%	*n*	%	*n*	%
Sex (*n*, %)	80, 34·6		26, 31·7		25, 30·9		29, 42·6	
Women	80	34·6	26	31·7	25	30·9	29	42·6
Men	151	65·4	56	68·3	56	69·1	39	57·4
	Mean	sd	Mean	sd	Mean	sd	Mean	sd
Weight (kg)	84	17·3	85·5	18·2	89·8	16·9	75·3	12·6^*^
BMI (kg/m^2^)	30·7	5·8	31·2	5·9	31·7	6·1	28·7	4·8^*^
NUTRIC score	3·5	1·5	3·4	1·7	3·5	1·3	3·7	1·2
SOFA	9·0	2·8	9·4	3·9	8·8	2·0	9·0	2·1
APACHE II	18·6	5·5	18·5	7·0	19·2	4·5	17·9	4·4
PAFI	103·7	45·2	117·8	62·7	93·5	22·5	98·9	35·4
	*n*	%	*n*	%	*n*	%	*n*	%
Pre-existing conditions (*n*, %)								
Overweight/obesity	200	86·6	71	86·6	75	92·5	54	79·4
Hypertension	77	33·3	32	39·0	26	32·0	35	51·4
Type 2 diabetes	84	36·3	23	28·0	26	32·0	35	51·4
Hypothyroidism	8	3·4	2	2·4	5	6·1	1	1·5
COPD	2	0·8	1	1·2	1	1·2	0	

IMM, immune-modulating enteral formula; ω3, enteral formula with ω3 fatty acids; MD, maltodextrin + glutamine; NUTRIC score, nutrition risk in the critically ill; SOFA, Sequential Organ Failure Assessment; APACHE II, Acute Physiology and Chronic Health Evaluation. PAFI, index PaO2/FiO2. Data shown as percentage, mean (standard deviation) and median.

* *P* < 0·05.

Enteral nutrition was started during the first 24 h after admission to the ICU. The mean calories prescribed were 22·5 (sd 3·3) kcal/kg per d (95 % CI 22·1, 22·9), and the mean protein prescribed were 1·7 (sd 0·3) g/kg per d (95 % CI 1·5, 1·6). A total of 7·7 % (*n* 18) patients received PN during their stay in addition to EN. The decision to combine them was mostly based on the presence of digestive tract bleeding, frequent in the IMM group compared with the ω3 and MD groups (*P* < 0·05). Patients in the ω3 group showed low levels of gastric residue compared with the other two groups (*P* = 0·001). The characteristics of the EN contributions are shown in Table 2.

**Table 2.** Characteristics and tolerance of enteral nutrition (Numbers and percentages; mean values and standard deviations)

Characteristics	Total		IMM group		ω3 group		MD group		*P*
	*n* 231		*n* 82		*n* 81		*n* 68		
	*n*	%	*n*	%	*n*	%	*n*	%	
Started enteral nutrition within 24 h, *n* (%)	231	100	82	100	81	100	68	100	0·191
	Mean	sd	Mean	sd	Mean	sd	Mean	sd	
Calories prescribed kcal/kg per d	22·5	3·3	22·5	3·5	22·2	3·0	23·0	2·2	0·283
Protein prescribed g/kg per d	1·7	0·3	1·7	0·3	1·7	0·3	1·7	0·2	0·210
Percent goal kcal day 3	92·9	16·6	81·9	23·3	99·5	3·6	98·1	7·1	0·001*
Percentage goal protein day 3	88·4	18·3	98·5	17·7	90·3	12·4	73·9	15·4	0·001*
	*n*	%	*n*	%	*n*	%	*n*	%	
≥ 80 % of Total caloric target day 3, *n* (%)	200	86·5	54	65·8	80	98·7	66	97	0·001*
≥ 80 % of Total protein target day 3, *n* (%)	157	67·9	69	84·1	63	77·7	25	36·7	0·001*
≥ 80 % Total caloric and protein target day 3, *n* (%)	135	58·4	49	59·7	62	76·5	24	35·2	0·001*
	Mean	sd	Mean	sd	Mean	sd	Mean	sd	
High gastric residual volume	513·8	341·2	527·1	343·4	395·8	304·1^†^	638·4	337·5	0·001^†^
	*n*	%	*n*	%	*n*	%	*n*	%	
Received parenteral nutrition, *n* (%)	18	7·7	9	10·9^‡^	0		9	13·2^‡^	0·01^‡^

IMM, immune-modulating enteral formula; ω3, enteral formula with ω3 fatty acids; MD, maltodextrin + glutamine. Data shown as percentage, mean (standard deviation) and median.

*All *P* < 0·001, ^†^*P* < 0·001 compared with IMM and MD, ^‡^*P*< 0·01 compared with ω3.

### Nutrition provision

#### Primary outcome

The percentage of patients achieving the primary outcome was significantly higher in the ω3 group (76·5 % *v*. 59·7 and 35·2 %, *P* < 0·001) compared with the IMM and MD groups, respectively. Patients achieved a mean of 92·9 % (s d 16·6; 95 % CI 90·7, 95·0) of the caloric target during the first 72 h with statistically significant differences between groups, with the ω3 group achieving the highest value (*P* < 0·001). Participants achieved a mean of 88·4 % (sd 18·3; 95 % CI, 86·3, 90·2) of protein target (g/d) with significant differences between groups, with IMM having the highest value (*P* = 0·001) (Table 2).

#### Secondary outcomes

**Figure 2.**
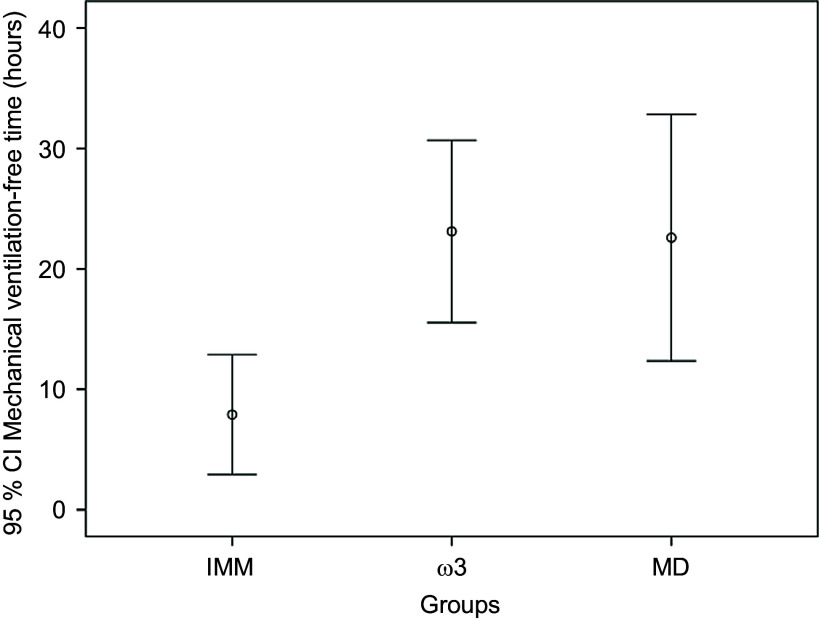
The mechanical ventilation-free time according to different enteral formulae.

Mechanical ventilation-free time was significantly longer in the ω3 and MD groups: 23·11 (sd 34·2) h (95 % CI 15·5, 30·68) and 22·59 (sd 42·2) h (95 % CI 12·36, 32·82), respectively, compared with the IMM group, which had 7·9 (sd 22·6) h (95 % CI 2·93, 12·88) (*P* < 0·01) (Fig. 2). The ICU mortality was similar in the three groups (53·6 *v*. 59·2, 69·1 %, *P* = 0·153).

Regarding markers of nutritional status and compared with baseline values, the IMM group showed a statistically significant increase in lymphocytes at the end of follow-up, the ω3 group showed differences in lymphocytes, prealbumin and transferrin levels and the MD group also in lymphocytes (all *P* < 0·05) (Table 3). Specifically, the mean final prealbumin observed was 0·203 ± 0·108 g/L and 0·203 ± 0·095 g/L in IMM and ω3 groups vs 0·164 ± 0·070 g/L (*p* < 0·01) MD group. In the case of transferrin, the mean final values were 1·515 ± 0·536 g/L and 1·521 ± 0·500 g/L in IMM and ω3 groups compared with 1·337 ± 0·483 g/L (*p* < 0·05) MD group was calculated. The increase of lymphocytes was particularly remarkable in the ω3 group 1056·7 (sd 660·8) cells/mm^3^ compared with 853·3 (sd 435·9) cells/mm^3^ and 942·7 (sd 675·4) cells/mm^3^ (*P* < 0·001) in the IMM and MD groups. Although all three groups showed a change in markers of nutritional status levels, the deltas of change by groups are shown in Table 3.

**Table 3.** Laboratory tests. Measurement of markers of nutritional status during follow-up

Characteristics	IMM group					ω3 group					MD group				
	*n* 82					*n* 81					*n* 68				
	Baseline		Final		Δ	Baseline		Final		Δ	Baseline		Final		Δ
Lymphocytes (cells/mm^3^)	831·1	784·5	853	435·9*	22·8	645·9	367·8	1056·7	660·8^*†††‡^	410·8	610·5	378·5	942·7	675·4*	332·2
Albumin (g/L)	29	4	26	4^*††^	3	30	3	27	5^*††^	3	30	4·3	26	5*	4
Prealbumin (g/L)	0·216	0·11	0·203	0·108*	0·013	0·195	0·097	0·203	095*	0·008	0·171	0·071	0·164	0·07*	0·007
Transferrin(g/L)	1·643	0·05	1·515	0·536^*†^	0·128	1·501	0·389	1·521	0·5^*†^	0·02	1·519	0·485	1·337	0·483*	0·182

IMM, immune-modulating enteral formula; ω3, enteral formula with ω3 fatty acids; MD, maltodextrin + glutamine. Data shown as percentage and median. Δ change (delta) scores.

*P < 0·05, **P < 0·01, ***P < 0·001, ^‡^*P* < 0·001 compared with IMM, ^†^*P* < 0·05 compared with MD, ^††^ *P* < 0·01 compared with MD, ^†††^*P* < 0·001.

### Gastrointestinal complications and tolerance

Gastrointestinal complications were observed in 12·5 % of participants. The most frequent was diarrhoea and gastrointestinal bleeding in 5·1 % (*n* 12) of both cases, which led to a reduction in the infusion rate of EN or temporary suspension (Table 4). Eighteen patients required total PN, with a median duration of 7·6 (sd 4·6) days. Statistically significant differences were observed in the tolerance of EN. The percentage of diarrhoea observed was lower in the ω3 group (1·2 %) compared with 4·8 and 10·2 % (*P* = 0·003) in the IMM and MD groups, respectively. Gastroparesis occurred in 4·8 and 1·2 % (*P* = 0·003) in the IMM and ω3 groups, respectively, compared with the MD group (*P* = 0·001).

**Table 4.** Gastrointestinal complications (Numbers and percentages)

Characteristics	Total		IMM group		ω3 group		MD group	
	*n* 231		*n* 82		*n* 82		*n* 68	
	*n*	%	*n*	%	*n*	%	*n*	%
General complications, *n* (%)	29	12·5	17	20·7	3	3·6^*^	9	13·2
GIB, *n* (%)	12	5·1	9	10·9	1	1·2	2	2·9
Diarrhoea, *n* (%)	12	5·1	4	4·8	1	1·2	7	10·2
Gastroparesis, *n* (%)	8	3·4	4	4·8	1	1·2	0	

IMM, immune-modulating enteral formula; ω3, enteral formula with ω3 fatty acids; MD, maltodextrin + glutamine; GIB, gastrointestinal bleeding.

* *P* < 0·05 compared with IMM and MD.

## Discussion

Adequate nutritional support for critically ill patients with SARS-Cov-2 infection was challenging for several reasons, especially during the early stages of the pandemic. According to the results of our study, patients admitted to the ICU had important risk factors: 87·9 % of them were overweight or had some degree of obesity, and more than 30 % had chronic diseases such as hypertension and diabetes. These co-morbidities are associated with poorer disease prognosis of the disease and have been linked to a significant deterioration in nutritional status during hospitalisation, despite admission with low nutritional risk according to the mNUTRIC score.^(2,18)^

The recommendations given so far for the nutritional support of critically ill patients with SARS-CoV-2 infection suggest that the use of high-protein and iso-osmolar polymeric formulas is safe and well tolerated.^(10,19)^ There really are no unified recommendations regarding the use of formulas with components that have shown certain benefits in other diseases, as is the case of formulas supplemented with ω3 fatty acids or formulas known as immunomodulators. However, the results of this study show that the use of enteral formulas supplemented with ω3 fatty acids, which have the characteristic of a higher energy density and high protein, facilitates achieving the caloric and protein requirements of patients. In a study by Doaei *et al*.,^(20)^ the addition of 0·4 g of EPA and 0·2 g of DHA to an enteral formula was associated with better renal and respiratory function and longer survival compared with critically ill control patients with COVID-19. On the other hand, a recent systematic review by Mazidimoradi *et al*.^(21)^ concluded that ω3 fatty acid deficiency is associated with greater mortality and severity of disease in critically ill patients with COVID-19. In our results, we do observe the benefits of the formula supplemented with ω3 fatty acids compared with other formulas in providing 80 % of the caloric and protein requirements. In addition, we observed that the mechanical ventilation-free time was significantly longer. Secondarily, this formula was associated with improvement in some markers of nutritional status. This effect may have been due to it being a high-calorie and high-protein formula that facilitated meeting the requirements of patients with lower volume and better tolerance during prone positioning.

As for the IMM formula, higher levels of lymphocytes were recorded compared with the other groups. However, these values were lower than the change observed in the group supplemented with ω3. The evidence of the effect of IMM formulas in critically ill patients with COVID-19 is much more limited, and studies have found few benefits when using this type of formula, as demonstrated by the clinical trial of Pimentel *et al*.,^(22)^ which reported a decrease in the levels of C-reactive protein in patients receiving an IMM formula, with no significant effect on any other variable. A pilot study by Scarcella *et al*. observed that immune nutrition prevented malnutrition development with a significant decrease of inflammatory markers in overweight patients admitted to the semi-intensive COVID-19.^(23)^

In the group that received the formula with MD plus glutamine, we observed a lower intake of calories and proteins compared with other groups. As for the secondary outcomes, a significantly longer ventilator-free time was observed, similar to the supplemented with the ω3 group. Lower levels of prealbumin, albumin and transferrin were found at the end of follow-up compared with the other groups. This may be related to the fact that the formula contained a lower amount of protein, and despite being added with a protein module, it was more complex to meet both the energy and protein requirements.

Early initiation of EN is recommended because of the reduced length of ICU stay, time on mechanical ventilation and the presence of complications.^(6,10,24)^ However, as shown by Farina *et al*.,^(25)^ the evidence for this benefit in critically ill patients with COVID-19 is inconclusive. In our study, all patients started EN within the first 24 h of ICU admission, eight out of ten patients met their energy requirements within the first 72 h and seven out of ten patients met their protein requirements within the same period. This study found an association between these variables and a reduction in mechanical ventilation-free time.

Despite the early initiation of EN in our study, the infusion rate was frequently reduced due to the presence of gastrointestinal complications, deterioration of the patient’s general condition or the use of prone positioning. In a systematic review by Bruni *et al*.,^(26)^ they concluded that patients in the prone position showed a higher incidence of EN discontinuation and vomiting episodes, but without changes in mechanical ventilation-free time, length of stay or mortality compared with patients in the supine position. On the other hand, Ellis *et al*.^(27)^ concluded in a review that the use of EN in the prone position is comparable to that in the supine position, without a greater risk of complications and safe in ventilated patients with COVID-19. In our experience, we agree with Behrens *et al*.^(28)^ who propose that the use of EN should not be contraindicated in patients in the prone position, nor should it affect the infusion rate or be an indication for the use of PN. Therefore, we suggest that the knowledge of the different members of the healthcare team regarding the safety of EN administration in the prone position should be strengthened, in order to avoid modifying or suspending the intervention, which could be detrimental to the patient’s clinical condition, due to non-compliance with the established caloric and protein targets.

Finally, gastrointestinal complications represent a significant challenge in the context of daily medical practice, with the potential to impact compliance with nutritional targets in patients diagnosed with COVID-19. A higher prevalence of diarrhoea was observed in the MD group (5·1 % *v*. 3·6 %), which may be attributed to the increased fibre content of these enteral formulas. This may also have contributed to the difficulty in achieving the set goals. In cases of an increased number of complications, EN is reduced or initiated to PN. The low incidence of complications lends support to the observation that EN in critically ill patients with SARS-CoV-2 infection is well tolerated, has a low incidence of associated complications and represents an excellent strategy to ensure nutritional support for patients with this pathology in the ICU.

This study has some limitations. First, our study was conducted during the initial waves of the SARS-CoV-2 pandemic, a period during which there was a lack of experience in managing the disease Second, the published data are derived from a single referral hospital centre, which may introduce selection biases, given that outside the hospital unit, there is limited access to the enteral formulas. Third, our unit has a standardised protocol for the initiation and progression of EN infusion, and it is uncertain that the same results would be observed using a different protocol. Fourth, the percentage of diarrhoea and gastroparesis reported can be different from that reported in other studies; this could be explained by the heterogeneity in the definition of diarrhoea and gastroparesis.

### Conclusions

In conclusion, the present study demonstrated a positive effect when using a high-calorie, high-protein formula with ω3 fatty acids to achieve calorie and protein targets. Second, a reduction in mechanical ventilation-free time was observed, which was associated with a lower deterioration of albumin levels and improved levels of lymphocytes, prealbumin and transferrin. Finally, we consider that those patients with critical illness due to SARS-CoV-2 should receive formulas that ensure an adequate supply of energy and nutrients, in accordance with international recommendations. It is therefore essential to consider the characteristics of the population in question when selecting an appropriate formula.
